# Targeted silencing of Jab1/Csn5 in human cells downregulates SCF activity through reduction of F-box protein levels

**DOI:** 10.1186/1471-2091-7-1

**Published:** 2006-01-09

**Authors:** Gregory A Cope, Raymond J Deshaies

**Affiliations:** 1 Department of Biology, California Institute of Technology Pasadena, CA 91125, USA; 2 Howard Hughes Medical Institute, 4000 Jones Bridge Road, Chevy Chase, MD, 20815-6789, USA; 3 Department of Biology, Stanford University, Palo Alto, CA 94305, USA

## Abstract

**Background:**

SCF ubiquitin ligases target numerous proteins for ubiquitin dependent proteolysis, including p27 and cyclin E. SCF and other cullin-RING ligases (CRLs) are regulated by the ubiquitin-like protein Nedd8 that covalently modifies the cullin subunit. The removal of Nedd8 is catalyzed by the Jab1/MPN domain metalloenzyme (JAMM) motif within the Csn5 subunit of the Cop9 Signalosome.

**Results:**

Here, we conditionally knock down Csn5 expression in HEK293 human cells using a doxycycline-inducible shRNA system. Cullin levels were not altered in CSN-deficient human cells, but the levels of multiple F-box proteins were decreased. Molecular analysis indicates that this decrease was due to increased Cul1- and proteasome-dependent turnover. Diminished F-box levels resulted in reduced SCF activity, as evidenced by accumulation of two substrates of the F-box protein Fbw7, cyclin E and c-myc, in Csn5-depleted cells.

**Conclusion:**

We propose that deneddylation of Cul1 is required to sustain optimal activity of SCF ubiquitin ligases by repressing 'autoubiquitination' of F-box proteins within SCF complexes, thereby rescuing them from premature degradation.

## Background

Proteins are marked for degradation by the 26S proteasome via the covalent attachment of chains of the 76-amino acid protein ubiquitin [reviewed in [1]]. This process involves three discreet steps. First, ubiquitin is activated by the ubiquitin conjugating enzyme (E1) through the hydrolysis of ATP to AMP to yield a high energy thioester intermediate between the C-terminal glycine of ubiquitin and the catalytic cysteine of the E1.

Subsequently, ubiquitin is transferred onto the catalytic cysteine of one of many ubiquitin conjugating enzymes (E2) which, in turn, transfer their cargo onto substrates with the help of ubiquitin ligase enzymes (E3).

One of the best-studied E3 ubiquitin ligase enzymes is the four subunit complex SCF [reviewed in [2]]. SCF consists of two activities: the first, contained within the Cul1 and RING domain Hrt1/Roc1/Rbx1 proteins, is the ability to recruit and activate the E2 to facilitate ubiquitin transfer from the E2 onto substrate; the second resides within the variable F-box proteins, which are linked to Cul1 via Skp1 and are thought to recruit substrates for ubiquitination by the Cul1/Hrt1 sub-complex. The large number of different F-box proteins gives SCF the opportunity to access a wide array of substrates. In yeast, over 19 F-box proteins are known, in *A. thaliana *over 400, and in humans ~70 [2]. The family of SCF ligases in turn is the prototype for a superfamily of cullin-RING ligases that, like SCF, are modular enzymes comprising a cullin-RING subcomplex linked to a variable substrate receptor subunit (VHL box proteins for Cul2, BTB proteins for Cul3, and SOCS box proteins for Cul5). Altogether, the human genome may have the capacity to code for as many as 350 different CRLs.

Given the diversity of CRL substrate receptor proteins, two important questions emerge. First, how is the repertoire of CRLs dynamically controlled? Second, are distinct CRL complexes differentially regulated in a manner that depends on the identity of the substrate receptor? One partial answer to both of these questions is that F-box and other substrate receptors are often unstable proteins, and it is thought that they are targeted for degradation in part by 'autoubiquitination' within SCF-E2 complexes [2]. However, not all CRL substrate receptors are unstable, and thus there must be some means of differentially controlling their stability. There are multiple ways in which this might be accomplished. First, CRL ubiquitin ligase activity is negatively regulated by Cop9 Signalosome (CSN) *in vitro *[3-6]. CSN cleaves the ubiquitin-like protein Nedd8 from the cullin subunit of CRLs [3,7]. Attachment of Nedd8 to Cul1 strongly stimulates the ability of the Cul1-Hrt1/Roc1/Rbx1 catalytic core to promote ubiquitin chain synthesis by Cdc34 E2 enzyme [8-10]. Once Nedd8 is detached, CAND1 can bind Cul1 and displace Skp1, thereby preventing the recruitment of substrate to the catalytic core [11,12]. In addition to removing Nedd8, CSN also recruits a deubiquitinating enzyme to Cul1, Ubp12, that opposes ubiquitin polymerization [6,13]. Thus, CSN may play a key role in controlling the dynamics of individual CRL complexes and the overall repertoire of different CRL complexes in a cell.

CSN is a highly conserved protein complex found from yeast to humans. CSN is composed of eight subunits, termed Csn1-Csn8 [14] and each of these subunits contains high homology to components of the 26S proteasome lid subcomplex and eukaryotic Initiation Factor 3 (eIF3) [reviewed in [15]]. CSN has been found to play diverse roles in several different organisms [reviewed in [15]]. In *A. thaliana*, CSN components were identified in a screen for plants that displayed a constitutive photomorphogenic defect (plants develop in the dark as they would in the light). In *D. melanogaster*, mutations in Csn4 and Csn5 result in pleitropic effects, including activation of meiotic checkpoints [16,17] and failure of photoreceptor neurons to differentiate [18]. RNAi of Csn5 in *C. elegans *additionally results in pleitropic effects, including sterile worms and alterations in microtubules [19].

Although the molecular basis behind many of these phenotypes has yet to be elucidated, it is becoming evident that deneddylation of cullins catalyzed by the 'JAMM' metalloprotease active site motif in the Csn5 subunit is at least partially responsible. Transgenic *csn5 *with mutations in the JAMM motif fails to correct the developmental defects of *csn5*-delete flies [20]. Moreover, failure to deneddylate Cul3 in *C. elegans *is implicated in accumulation of the microtubule severing protein Mei-1, which results in microtubule defects [19,21].

The effects of loss of deneddylation of cullin proteins are still not understood. Loss of CSN function causes a defect in cullin-based ligase activity *in vivo *suggesting that deneddylation promotes SCF activity [4,7,20-25]. However, *in vitro *data suggests a negative role for CSN in regulation of SCF [3-6]. What may account for this discrepancy? One hypothesis suggests that neddylation and deneddylation affect cycles of assembly of CRL complexes and the stability of substrate adaptor proteins *in vivo *[15,6].

In an effort to investigate how loss of deneddylation affects SCF activity, we conditionally silenced the catalytic subunit of CSN in mammalian cells. Suppression of Csn5 protein resulted in a significant decrease in the F-box proteins Skp2, cyclin F, Fbx7, Fbx4, and Fbw7. Moreover, mRNA transcripts for all but one of these F-box proteins were unaltered when *CSN5 *was suppressed, suggesting the decreases in protein levels are post-translational. Consistent with this notion, treatment with proteasome inhibitors largely restored the levels of multiple F-box proteins and dominant-negative Cul1 prevented loss of cyclin F. Finally, we found a dramatic increase in the protein and activity levels of the SCF^Fbw7 ^substrate cyclin E, suggesting that loss of F-box proteins in CSN-deficient cells results in substrate accumulation.

## Results

### Depletion of CSN5 in HEK293 cells is not lethal

**Figure 1:**
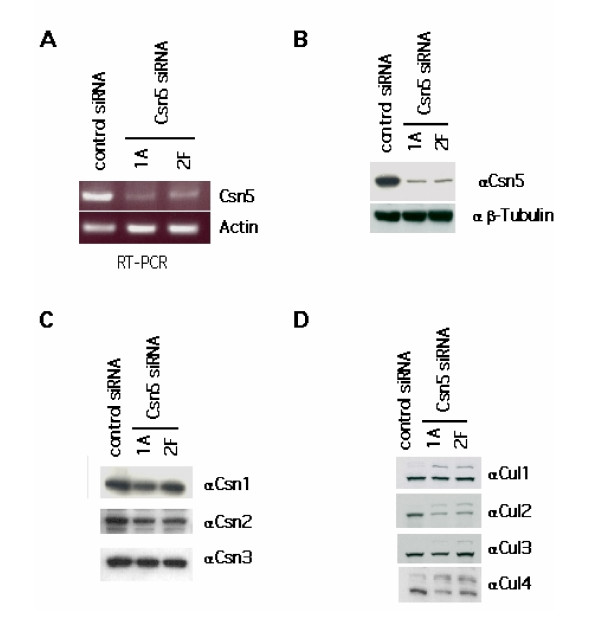
**Suppression of Csn5 in HEK 293 human cells. (A) **Expression of Csn5-specific shRNA suppresses endogenous Csn5 mRNA levels. Cells induced with doxycycline for 8 days were harvested and analyzed by RT-PCR using oligos specific for Csn5. **(B) **Expression of Csn5-specific shRNA suppresses endogenous Csn5 protein levels. Cells induced with doxycycline for 8 days were harvested and lysed. Lysates were analyzed by western blot using antibodies specific toward human Csn5. **(C) **Silencing of Csn5 does not appreciably affect the levels of other CSN subunits. Cells induced with doxycycline for 8 days were harvested, and cell lysates were analyzed by western with the antibodies indicated. **(D) **Silencing of Csn5 results in altered cullin neddylation. Cells were induced with doxycycline for 8 days and harvested. Cells were lysed and analyzed by western blot using the antibodies indicated.

To analyze the effect of loss of deneddylation in human cells, we utilized the doxycyline-inducible shRNA system developed by Clevers and colleagues [26] to conditionally down-regulate Csn5 protein levels. Eight days of doxycycline treatment of cells carrying the inducible Csn5-specific shRNA resulted in a drastic reduction in both Csn5 mRNA and protein levels when compared to induced cells expressing a scrambled shRNA (Figures 1A and 1B). By contrast, the levels of other CSN subunits showed little or no change (Figure 1C). Despite this significant drop in Csn5 protein levels, few morphological changes were observed, the cells continued to grow and divide, and little change in steady-state cell cycle distribution was detected by FACS (data not shown). This is consistent with prior observations on normal diploid BJ1 fibroblasts depleted of Csn5 [4]. Given that loss of CSN function results in accumulation of the neddylated form of cullins in yeast, *Drosophila*, and *C. elegans*, we analyzed the endogenous cullins 1–4 by western blot in human cells depleted of Csn5. Loss of Csn5 resulted in enhanced neddylation of all four cullins, although to varying extents (Figure 1D). The failure to observe complete conversion of all cullins to the neddylated species – as was seen for Cul1 in *csn1*Δ *S. pombe *cells [3] – may be due to residual Csn5 activity, an alternative cullin deneddylase, limiting Nedd8 attachment activity, or the sequestration of deneddylated cullins into complexes with CAND1 [12]. Despite the change in Nedd8 modification, the total levels of cullins 1–4 were largely unaltered in Csn5-depeleted human cells, except for a modest reduction in the level of Cul2.

### Loss of deneddylation causes a reduction in F-box protein levels

**Figure 2:**
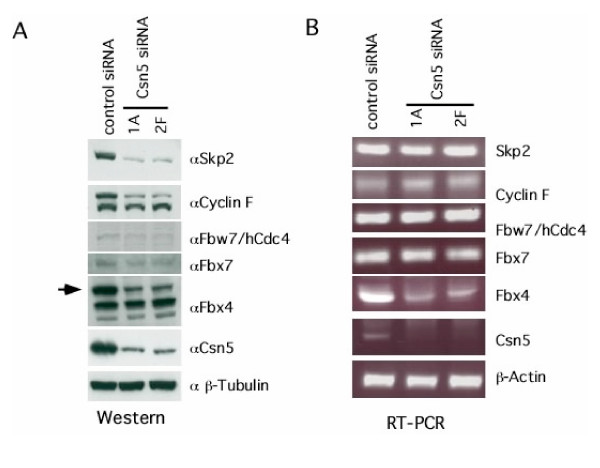
**Loss of Csn5 results in a decrease in F-box protein levels. (A) **Depletion of Csn5 results in a decrease in F-box protein levels. Cells were treated with doxycycline for eight days followed by lysis and SDS-PAGE/western blot analysis with antibodies toward the proteins indicated. **(B) **Depletion of Csn5 does not affect F-box protein mRNA levels. Cells were treated with doxycycline for eight days and analyzed by RT-PCT using oligos specific toward the mRNAs indicated.

It has been proposed that loss of deneddylation could render F-box adaptor proteins unstable [6,15]. To test this hypothesis, we analyzed the levels of several endogenous F-box proteins in cells depleted of Csn5. In this experiment and throughout this report, we focused our effort on endogenous proteins to avoid potential artifacts associated with overexpression of recombinant proteins. Concomitant with loss of Csn5, we observed significant decreases in the steady-state protein levels of Skp2, cyclin F, Fbw7, Fbx4 and Fbx7 (Figure 2A). To test whether this decrease in protein levels was post-transcriptional, we performed RT-PCR on the corresponding mRNA transcripts. Despite the significant drop in protein levels, there was little change in mRNA levels, suggesting that for 4 of the 5 F-box proteins examined (Fbx4 being the exception) the loss of protein was due to reduced translation or increased degradation (Figure 2B). Interestingly, two F-box proteins, β-TrCP and Emi1, showed little or no change in protein level when Csn5 was suppressed (data not shown).

**Figure 3:**
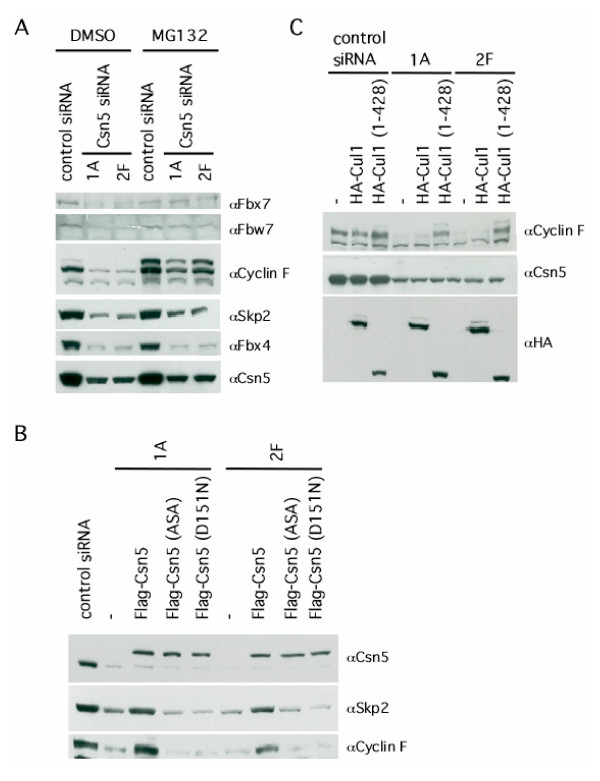
**Loss of Csn5 results in proteasome-dependent turnover of F-box proteins. (A) **Supplementation of doxycycline-treated cells with MG132 inhibits the turnover of F-box proteins. Cells were treated with doxycycline for eight days followed by treatment with MG132 (25 *μ*M) for eight hours. Cells were lysed in SDS and analyzed by SDS-PAGE/western blot for the indicated proteins. **(B) **Ectopic expression of wild-type, but not JAMM point mutant Csn5, can rescue the decrease in protein levels of Skp2 and cyclin F. Cells were treated with doxycycline for six days, followed by transient transfection with wild-type Flag-Csn5, Flag-Csn5 (ASA), or Flag-Csn5 (D151N). Forty-eight hours post-transfection, cells were lysed in SDS and analyzed by SDS-PAGE/western blot for the indicated proteins. **(C) **Ectopic expression of a dominant-negative Cul1 restores cyclin F protein levels. Control, wild-type, or Cul1 (1–428) expression plasmids were transfected into cells induced with doxycycline for six days. Forty-eight hours post-transfection, cells were lysed in SDS and analyzed by SDS-PAGE/western blot with antibodies against the indicated proteins.

To determine if the decrease in F-box protein levels was specific and due to loss of JAMM isopeptidase activity, we ectopically expressed mouse Csn5 in cells depleted of endogenous Csn5 by shRNA. Transfection of wild-type mouse *CSN5 *cDNA, which differs by two nucleotides from human *CSN5 *in the region that is targeted by the silencing shRNA, restored wild-type levels of both Skp2 and cyclin F (Figure 3B). In contrast, expression of two different JAMM point mutants of mouse Csn5 failed to restore Skp2 and cyclin F protein levels, despite normal levels of expression of the mutant proteins (Figure 3B). The protein levels of Fbx4 could not be rescued by overexpression of wild-type mouse *CSN5*, suggesting that loss of Fbx4 arose from a promiscuous effect of the *CSN5*-directed siRNA and was not due to Csn5 knockdown (data not shown), Therefore, the reduction in Skp2 and cyclin F levels in Csn5-depleted cells is caused by the loss of CSN isopeptidase activity.

### Loss of F-box proteins is dependent on the ubiquitin-proteasome system

We next examined whether the 26S proteasome was involved in the reduction of F-box protein levels. Treatment of Csn5-depleted cells with the proteasome inhibitor MG132 largely restored normal levels of Fbx7, cyclin F, and Fbw7, whereas Skp2 levels were only partially rescued (Figure 3A). By contrast, the loss of Fbx4 was not reversed by proteasome inhibition, which is consistent with our prior observation that Csn5 shRNA non-specifically diminished the levels of Fbx4 transcripts. Similar results were obtained for all proteins when we used the proteasome inhibitor LLnL. Therefore, the loss of F-box proteins in Csn5-depleted cells appears to depend largely upon the 26S proteasome.

The role of the proteasome in mediating the loss of F-box proteins in Csn5-depleted cells suggested that Csn5 might normally protect F-box proteins within SCF complexes from being 'autoubiquitinated' by the associated E2 enzyme and then degraded. To address this possibility we sought to examine the levels of a representative F-box protein in cells in which Csn5 was depleted and SCF activity was simultaneously inhibited. A C-terminal truncation mutant of Cul1 [Cul1 (1–428)] possesses dominant-negative activity [27]. To determine if the loss of cyclin F in Csn5-depleted cells was SCF dependent, we transiently expressed either wild-type or Cul1 (1–428) in doxycycline-induced cells. Whereas expression of full length Cul1 did not alter cyclin F accumulation, expression of Cul1 (1–428) restored cyclin F to levels comparable to those seen in untreated cells (Figure 3C). These data indicate that loss of cyclin F was dependent upon an intact SCF complex.

### Loss of Fbw7 results in a marked increase in substrate levels

**Figure 4:**
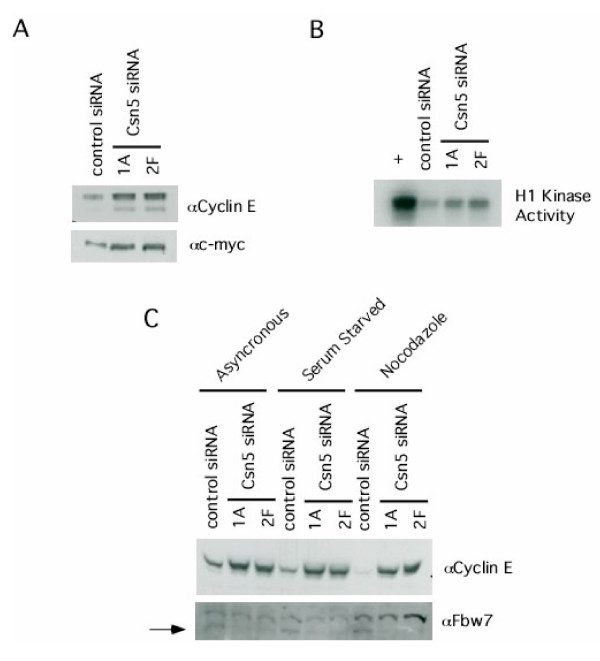
**Loss of Csn5 results in an increase in cyclin E, an F-box protein substrate. (A) **Substrates of the Fbw7 SCF complex accumulate when Csn5 is depleted. Cells were induced with doxycycline for eight days followed by SDS-PAGE/western blot analysis for the indicated proteins. **(B) **Activity of the protein kinase cyclin E/Cdk2 is increased when cells are depleted for Csn5. Cells were induced with doxycycline for eight days followed by lysis under native conditions. Lysates were immunoprecipitated with antibodies toward cyclin E and immuoprecipitates analyzed for kinase activity against histone H1. **(C) **Cell cycle regulation of cyclin E is lost when cells are depleted for Csn5. Cells were induced with doxycycline for eight days followed by arrest at the indicated cell cycle stages. Cell lysates were analyzed by SDS-PAGE/western blot with the indicated antibodies.

Given the reduction in Fbw7 levels in Csn5-depleted cells, we analyzed two substrates of SCF^Fbw7 ^– cyclin E and c-myc – to see if they were stabilized. Down-regulation of Csn5 resulted in accumulation of both c-myc and cyclin E (Figure 4A) as well as cyclin E-associated histone H1 kinase activity (Figure 4B). Moreover, depletion of Csn5 resulted in a loss of cell cycle regulation of cyclin E protein abundance (Figure 4C), which is consistent with a defect in cyclin E turnover.

Overexpression of a non-degradable form of cyclin E results in chromosome instability [28]. To examine chromosome stability in Csn5-depleted cells, we induced cells for 21 days with doxycycline and examined them for chromosome abnormalities by FISH and karyotyping. Despite a significant increase in cyclin E, we could not detect any chromosomal abnormalities (data not shown).

## Discussion

CSN downregulates the activity of SCF ubiquitin ligase in biochemical reconstitution assays [3-6] whereas loss of CSN components in several different organisms causes a defect in SCF activity *in vivo *[4,7,20-25]. To understand the basis for these paradoxical observations, we investigated the nature of SCF complexes in cells deprived of CSN activity. Here, we report that suppression of Csn5 in human cells resulted in a decrease in steady-state F-box protein levels. This decrease was post-transcriptional, stemmed from loss of the metallopeptidase activity of Csn5, and was largely proteasome-dependent. For cyclin F, the reduction in levels was blocked by a dominant-negative mutant of Cul1. As a consequence of the decline in F-box protein levels, ligands that are targeted for degradation by these F-box proteins are expected to accumulate. Indeed, both cyclin E and c-myc, substrates of SCF^Fbw7^, accumulated in Csn5-depleted cells. Although our study focused on F-box substrate receptor subunits of SCF, we anticipate that depletion of Csn5 will have similar consequences for VHL box, BTB domain, and SOCS box proteins that deliver substrates to Cul2, Cul3, and Cul5, respectively.

Loss of Csn5 causes both loss of deneddylation activity and loss of CRL-associated deubiquitination activity mediated by the deubiquitinating enzyme Ubp12 [6]. Ubiquitination and turnover of CRL substrate receptor proteins is suppressed by Ubp12 [13], and thus it is possible that the loss of F-box proteins that we observed in Csn5-depleted cells was due to loss of either deneddylation by Csn5 and/or F-box protein deubiquitination by Ubp12. However, expression of Csn5 carrying point mutations in the JAMM motif did not restore cyclin F or Skp2 protein levels in cells depleted of endogenous Csn5 (Figure 3B), even though human CSN complexes bearing a mutated JAMM domain possess associated deubiquitinating activity equivalent to that of wild type CSN [4]. Thus, we propose that loss of CSN-dependent deneddylation is sufficient to bring about a reduction of steady-state F-box protein levels in human cells.

The exact mechanism by which the substrate adaptors are turned over in Csn5-depleted cells is unknown. However, the stimulatory effect of neddylation on SCF activity *in vitro *[3-6] and the restoration of cyclin F levels by dominant-negative Cul1 in Csn5-depleted cells suggests an autocatalytic mechanism wherein an F-box protein is ubiquitinated by the neddylated Cul1-Hrt1 catalytic module present in the same SCF complex. We propose that once an SCF ubiquitin ligase exhausts its pool of available substrate, deneddylation of the Cul1 subunit by CSN brings about disassembly of the complex, with the end result being that the Skp1-F-box heterodimer is released and Cul1-Hrt1 are sequestered into an inactive assemblage with CAND1. In Csn5-depleted cells, the inactivation process is not initiated and therefore the F-box protein remains constitutively associated with a highly active, neddylated cullin-RING catalytic core, resulting in a high rate of F-box protein ubiquitination and degradation.

Interestingly, whereas most F-box proteins that we examined (e.g. cyclin F) exhibited a significant decline in levels upon depletion of Csn5, others (β-TrCP, Emi1) were not affected (G.A.C., unpublished data). We do not understand the basis for this difference, but it is unlikely to reflect whether a given F-box protein is turned over in normal cells, because β-TrCP levels were not reduced in Csn5-depleted cells even though transfected β-TrCP is turned over with a reasonably brisk half-life of ~120 minutes [29]. Clearly, more work is needed to determine why accumulation of only some F-box proteins depends upon CSN.

Given the substantial reduction in the steady-state levels of multiple F-box proteins upon depletion of Csn5, it is surprising that depleted cells continued to proliferate with normal cell cycle kinetics and did not exhibit a dramatic phenotype. For example, overexpression of cyclin E [28] or deletion of Fbw7 [30] causes chromosomal instability, but we were unable to detect any measurable defect in chromosome status in Csn5-depleted cells (data not shown). Given that depletion of Csn5 can affect so many different F-box proteins and presumably SOCS/BC box and BTB domain proteins as well, it is possible that opposing pathways were perturbed in such a way as to prevent the onset of chromosome instability. A similarly mild growth effect of depleting Csn5 was seen in a previous study of the normal diploid human fiboroblast BJ1 cell line [4]. As we have noted previously, the function of Csn5 may be more critical in a multicellular context, or in the face of differentiation signals that evoke a major change in the repertoire of cellular proteins [15].

While this manuscript was in preparation, it was reported that loss of function mutations in CSN components in *S. pombe *and *Neurospora *renders the substrate-recruiting subunits of both Cul1 and Cul3-based CRL complexes unstable, resulting in accumulation of their respective targets [13,31]. In addition, deneddylation of cullins mediated by CSN is required for the maintenance of cullin protein levels in *Drosophila *and for Cul1 stability in *Neurospora *[31,32]. In our original studies in *S. pombe *[3], we observed normal levels of Cul1 in a *csn1*Δ mutant, and this result has been confirmed and extended to other cullins in *S. pombe *[6,13] as well as *Arabidopsis thaliana *[33]. The results reported here indicate that accumulation of human cullins does not depend on CSN-dependent deneddylation. It is unclear why human, fission yeast, and *Arabidopsis *cullins behave differently from those of *Drosophila *and *Neurospora*.

## Conclusion

We have shown that CSN can stabilize F-box proteins, thus acting positively on SCF. CSN has been shown to participate in several different processes, including development, transcription, and cell cycle progression [reviewed in [15]]. We propose that loss of deneddylation and a consequent decline in F-box protein abundance underlies these phenotypes.

## Methods

### Cell culture, cell lines and plasmids

HEK293 cell lines were obtained from ATCC. Cell Lines were grown in Minimum Essential Media (MEM) supplemented with 10% FBS and grown at 37C with 5% CO_2_. For G1 cell cycle arrest, cells were grown in MEM media without Serum for 24 hours. For G2 cell cycle arrest, cells were treated with 330 nM of Nocodazole (Sigma) for 24 hours. All transfections were preformed using the calcium phosphate method. Briefly, cells were grown to a density of 1.2–2.0 × 10^6 ^cells per 6 cm plate. 5 ug of DNA was mixed to a volume of 450 uL and 50 uL of 2.5 M CaCl_2 _was added. 500 uL of BBS (0.05 M BES, 0.28 M NaCl, 0.0015 M Na_2 _HPO_4_, pH 7.0) was added and solution vortexed to mix. Cells were re-fed with fresh MEM supplemented with 2.5 uL/mL 25-hydroxycholesterol (Sigma). Precipitate was applied to cells and incubated for 4 hours prior to removal with PBS. Cells were re-fed with fresh MEM and incubated 24–48 hours. All plasmids used in this study are listed in Table 1.

**Table 1:** Plasmids used in this study

**Plasmid**	**Reference**	**Description**
pGC95	This study	Tetracycline inducible scrambled siRNA
pGC93	This study	Tetracycline inducible siRNA to human Csn5.
RJD 1419	This study	CMV Flag-Csn5
RJD 1500	This study	CMV Flag-Csn5 (ASA)
RJD 1501	This study	CMV Flag-Csn5 (D151N)
RJD 942	[34]	HA-Cul1
RJD 1192	[27]	HA-Cul1 (1–498)

### Construction of stable cell lines

HEK293 cells were transfected with pGC95 (control scrambled shRNA, 5'-CGTGCAAGGTCAGTACATGTTCAAGAGACATGTACTGACCTTGCACG) or pGC93 (5'-TGCTCAGGCTGCTGCATATTTCAAGAGAATATGCAGCAGCCTGAGCA). 24 hours after transfection, cells were re-fed with MEM supplemented with 5 ug/mL puromycin and selected for 48 hours. Post-selection, cells were grown for 7–10 days and single colonies were selected. Two representative clonal expansions (1A and 2F) of pGC93 are shown.

### mRNA analysis

RNA was purified from cells using Qiagen RNeasy kits. After purification, RNA was normalized by concentration used as a template for stratascript reverse transcriptase following manufacturers protocols (stratagene). Reverse transcribed RNA was then used for PCR towards Csn5, β-Actin, cyclin F, Fbx4, Fbx7, Fbw7, and Skp2.

### Protein analysis

Native lysates were made by resuspending cell pellets in an equal volume of buffer A (25 mM TRIS (7.5), 150 mM NaCl, 0.3% Triton X-100, 1 mM EDTA and 1 mM DTT supplemented with 1 mM PMSF, 0.25 ug/mL pepstatin, and 5 ug/mL each of leupeptin, aprotinin, and chymotrypsin). Lysates were cleared by centrifugation and normalized using the Biorad Protein Assay (Biorad). Denatured lysates were made by resuspending cell pellets in an equal volume of boiling SDS buffer (2% SDS, 50 mM TRIS (7.5) and 5 mM DTT) and boiled for 5 minutes. Lysates were sonicated and cleared by centrifugation.

### Antibodies

The following antibodies were used in this study: Fbw7 (Orbigen PAB-10563), cyclin F (Santa Cruz Biotechnology sc-952), cyclin E (Santa Cruz Biotechnology sc-198), Csn5 (Santa Cruz Biotechnology sc-9074), β-tubulin (Santa Cruz Biotechnology sc-9104) Fbx7 (Zymed 51–8000) and c-myc (Santa Cruz Biotechnology sc-42). Skp2 was generously provided by W. Krek and Fbx4 was generously provided by W. Boelens.

### Histone H1 kinase assay

Native lysates were immunoprecipitated for 1 hour with antibodies toward cyclin E. Immunoprecipitates were washed 3 times in native lysis buffer (buffer A). Beads were resuspended in 10 uL of reaction mix (for 100 uL: 3 uL 5 mg/mL histone H1, 1.5 uL 4.5 mM ATP, 1.5 uL γ-^32^P ATP, 94 uL of kinase assay buffer (10 mM TRIS (7.5), 10 mM MgCl_2_, 50 mM NaCl, 2 mM EDTA, 1 mM DTT and 0.02% Triton X-100)). Reactions were incubated for 20 minutes at 21°C and analyzed by SDS-PAGE followed by autoradiography.

## Abbreviations

CSN: COP9/signalosome

SCF: SKP1/Cullin/F-box protein complex

JAMM: Jab1/MPN domain metalloenzyme motif

BTB: "bric-a-brac/tramtrack/broad" protein complex

## Authors' contributions

All experiments were performed by GC. Both GC and RJD conceived and designed the experiments. Both authors contributed to the writing of the manuscript and both authors read and approved the final manuscript.
